# Supplementary material for: Visualizing drug binding interactions using microcrystal electron diffraction
Source: Commun Biol. 2020 Jul 31;3:417. doi: 10.1038/s42003-020-01155-1 (PMC7395157; doi:10.1038/s42003-020-01155-1)
Supplement: Supplementary file 1 — Supplementary Information [file 42003_2020_1155_MOESM1_ESM.pdf]

Supplementary Information

Visualizing drug binding interactions using microcrystal electron diffraction

Clabbers *et al.*

Supplementary Table 1. Unit cell parameters and estimated standard deviations of individual native HCA II crystal datasets as reported by XDS[1]

Dataset	*a* (Å)	*b* (Å)	*c* (Å)	β (°)
1	42.63(4)	41.11(1)	73.29(17)	103.02(24)
2	42.51(3)	40.98(1)	73.55(28)	105.27(18)
3	42.44(5)	41.06(1)	71.49(6)	105.37(19)
4	42.45(6)	41.22(3)	73.12(36)	103.33(36)
5	43.01(14)	41.29(2)	73.52(38)	107.59(89)
6	42.42(3)	41.10(1)	70.81(12)	103.18(33)
7	42.71(4)	41.75(2)	70.58(2)	104.84(48)
8	42.54(3)	40.70(6)	73.10(2)	104.27(5)
9	43.60(12)	41.47(1)	74.79(41)	100.94(58)
10	42.36(4)	41.19(2)	70.98(21)	105.72(8)
11	42.22(5)	41.36(1)	72.78(16)	104.50(14)
12	42.44(5)	41.22(5)	71.97(2)	104.40(13)

Data were integrated using XDS with the Laue group constrained to 2/*m*. Values in parenthesis show estimated standard deviation in unit cell parameters.

Supplementary Table 2. Unit cell parameters and estimated standard deviations of individual HCA II:AZM crystal datasets as reported by XDS[1]

Dataset	*a* (Å)	*b* (Å)	*c* (Å)	β (°)
1	42.83(7)	42.59(20)	71.99(8)	105.65(9)
2	42.69(10)	42.29(7)	71.96(11)	104.68(14)
3	42.12(9)	41.73(5)	72.69(28)	106.83(12)
4	43.16(7)	41.87(3)	71.94(22)	102.34(13)
5	42.93(7)	42.04(11)	72.36(80)	106.75(11)
6	42.29(4)	41.79(7)	72.21(29)	106.18(13)
7	42.85(9)	42.03(9)	71.46(20)	103.33(13)
8	43.62(13)	41.67(5)	71.33(21)	103.09(30)
9	44.27(26)	41.58(4)	71.34(21)	104.22(39)
10	41.79(20)	41.21(1)	73.14(31)	106.56(31)
11	42.49(2)	41.59(1)	72.45(7)	104.88(5)
12	42.60(8)	41.71(6)	72.61(10)	103.52(7)
13	42.44(6)	41.67(7)	72.33(45)	103.42(15)

Data were integrated using XDS with the Laue group constrained to 2/*m*. Values in parenthesis show estimated standard deviation in unit cell parameters.

Supplementary Table 3. Data merging statistics listed per resolution bin for the native HCA II MicroED data as reported by XSCALE[1]

Resolution limit	Observed reflections	Unique reflections	Complete-ness (%)	R_{merge} (%)	R_{meas} (%) [2]	$I/\sigma I$	$CC_{1/2}$ (%) [3]
11.18	363	67	59.8	18.2	19.7	8.55	98.2
7.91	868	138	73.0	17.2	18.6	8.99	97.4
6.46	1158	175	72.9	21.1	22.7	7.73	95.0
5.59	1407	208	74.6	22.5	24.0	8.02	96.9
5.00	1516	233	74.7	19.3	20.8	8.40	98.1
4.56	1666	255	74.3	19.9	21.3	8.95	98.6
4.23	1865	274	75.5	19.8	21.2	8.67	98.4
3.95	2206	323	78.6	22.6	24.3	8.01	96.7
3.73	2115	320	76.2	25.2	26.9	7.60	95.6
3.54	2316	345	76.8	29.5	31.5	6.64	94.1
3.37	2521	364	75.8	33.3	35.6	5.78	91.3
3.23	2440	374	75.7	39.2	42.0	4.71	89.5
3.10	2465	379	77.0	43.3	46.3	4.23	89.4
2.99	2533	406	75.5	53.7	57.6	3.30	85.5
2.89	2474	423	75.9	58.0	62.4	2.96	78.5
2.80	2013	413	73.4	74.8	81.4	2.14	72.2
2.71	1869	405	71.1	72.1	79.2	1.97	69.7
2.64	1812	413	67.0	79.4	87.8	1.68	67.2
2.56	1714	402	65.5	88.0	98.2	1.48	59.7
2.50	1537	374	60.0	94.5	105.4	1.23	46.5
overall	36858	6291	72.6	28.5	30.6	4.81	97.6

Data were truncated at approximately $I/\sigma I \geq 1.0$ and $CC_{1/2} \geq 0.4$ with a correlation significant at the 0.1% level[3].

Supplementary Table 4. Data merging statistics per resolution bin for the HCA II:AZM MicroED data a reported by XSCALE[1]

Resolution limit	Observed reflections	Unique reflections	Complete-ness (%)	R_{merge} (%)	R_{meas} (%) [2]	$I/\sigma I$	$CC_{1/2}$ (%) [3]
11.18	297	73	65.2	15.8	18.2	8.20	96.9
7.91	672	149	79.3	16.1	18.3	7.81	96.0
6.46	929	189	79.1	23.0	25.9	6.45	93.5
5.59	1106	229	82.4	21.8	24.4	6.54	94.2
5.00	1236	253	82.7	18.8	21.0	7.54	96.5
4.56	1441	285	81.7	14.7	16.3	8.54	97.8
4.23	1470	297	81.1	17.9	20.0	8.40	97.2
3.95	1656	329	81.6	18.7	20.7	7.91	96.7
3.73	1768	343	81.7	21.8	24.2	7.32	95.6
3.54	1769	362	81.7	24.3	27.1	6.24	91.1
3.37	2005	394	82.9	31.5	35.1	5.39	90.1
3.23	2128	413	83.3	34.2	38.0	5.10	88.1
3.10	2070	414	81.8	39.9	44.4	4.27	86.6
2.99	2116	436	83.5	50.4	56.3	3.47	79.1
2.89	1998	451	82.3	54.9	61.7	3.12	76.6
2.80	1969	468	81.2	71.2	80.0	2.44	73.3
2.71	1735	467	81.8	67.5	76.5	2.36	63.5
2.64	1460	451	75.5	74.5	86.8	1.99	57.3
2.57	1400	463	74.3	88.1	103.1	1.71	61.2
2.50	1232	436	71.1	87.7	102.7	1.52	58.4
overall	30457	6902	80.0	27.2	30.5	4.66	95.9

Data were truncated at approximately $I/\sigma I \geq 1.0$ and $CC_{1/2} \geq 0.4$ with a correlation significant at the 0.1% level[3].

Supplementary References

1. Kabsch, W. XDS. *Acta Cryst. D* **66**, 125–132 (2010).
2. Diederichs, K. & Karplus, P. A. Improved R-factors for diffraction data analysis in macromolecular crystallography. *Nat. Struct. Biol.* **4**, 269–275 (1997).
3. Karplus, P. A. & Diederichs, K. Linking crystallographic model and data quality. *Science* **336**, 1030–1033 (2012).
